# Changes in network centrality of psychopathology symptoms between the COVID-19 outbreak and after peak

**DOI:** 10.1038/s41380-020-00881-6

**Published:** 2020-09-14

**Authors:** Yuanyuan Wang, Zhishan Hu, Yi Feng, Amanda Wilson, Runsen Chen

**Affiliations:** 1 National Clinical Research Center for Mental Disorders, Department of Psychiatry, and China National Technology Institute on Mental Disorders, The Second Xiangya Hospital of Central South University, Changsha, 410011 Hunan China; 2 Division of Psychology, Faculty of Health and Life Sciences, De Montfort University, Leicester, UK; 3 State Key Laboratory of Cognitive Neuroscience and Learning, Beijing Normal University, Beijing, China; 4 Mental Health Center, Central University of Finance and Economics, Beijing, China; 5 School of Psychology, Beijing Normal University, Beijing, China; 6 Department of Psychiatry, The Affiliated Brain Hospital of Nanjing Medical University, Nanjing, China; 7 Department of Psychiatry, University of Oxford, Oxford, UK

**Keywords:** Diseases, Psychiatric disorders

## Abstract

The current study investigated the mechanism and changes in psychopathology symptoms throughout the COVID-19 outbreak and after peak. Two studies were conducted separately in China during outbreak and the after peak stages, with 2540 participants were recruited from February 6 to 16, 2020, and 2543 participants were recruited from April 25 to May 5, 2020. The network models were created to explore the relationship between psychopathology symptoms both within and across anxiety and depression, with anxiety measured by the Generalized Anxiety Disorder-7 and depression measured by the Patient Health Questionnaire-9. Symptom network analysis was conducted to evaluate network and bridge centrality, and the network properties were compared between the outbreak and after peak. Noticeably, psychomotor symptoms such as impaired motor skills, restlessness, and inability to relax exhibited high centrality during the outbreak, which still relatively high but showed substantial remission during after peak stage (in terms of strength, betweenness, or bridge centrality). Meanwhile, symptoms of irritability (strength, betweenness, or bridge centrality) and loss of energy (bridge centrality) played an important role in the network after the peak of the pandemic. This study provides novel insights into the changes in central features during the different COVID-19 stages and highlights motor-related symptoms as bridge symptoms, which could activate the connection between anxiety and depression. The results revealed that restrictions on movement were associated with worsen in psychomotor symptoms, indicating that future psychological interventions should target motor-related symptoms as priority.

## Introduction

The COVID-19 pandemic has caused substantial threats to people’s physical health and lives, as well as triggered psychological distresses such as anxiety and depression [1]. Unlike previous infections, worldwide mass media reports have highlighted the unique threat of COVID-19, increasing people’s psychological distress and panic [2]. COVID-19 is considered highly contagious and currently there is no targeted medical treatment available, instead reducing exposure to the virus is considered to be the best prevention strategy [2]. However, the negative effects of COVID-19 on mental health could be exacerbated by prevention-related measures, such as social distancing and isolation, resulting in a continued fear and panic toward the virus [3]. Therefore, timely mental health care has been required during this pandemic [4]. In order to provide the general public with appropriate mental health care, researchers have made an urgent call for guidance and practical evidence to inform the creation of both health and psychological interventions [5]. A number of recent studies have focused on mental health problems during COVID-19, with the most frequently reported symptoms being depression and anxiety aspects [1, 6, 7]. A meta-analysis on the mental health within the general population during the COVID-19 pandemic reported the prevalence of anxiety to be 31.9% (95% CI: 27.5–36.7) and the prevalence of depression as 33.7% (95% CI: 27.5–40.6) [8]. When understanding mental health problems, co-occurrence becomes a complex and principal issue in regards to treatment adherence and engagement in prevention measures [9]. Considerations to better understand co-occurrence during the pandemic are required.

Depression and anxiety are commonly co-occur at high rates, with a co-occurrence of depression and anxiety resulting in more severe and chronic psychopathology [10, 11]. Several theoretical models have been proposed to explain the co-occurrence of anxiety and depression; the diathesis-stress model proposes a simultaneously development of symptoms and left untreated anxiety could increase the risk of depressive disorders and vice versa [12–15]. However, there is no universal agreement to explain the co-occurrence of anxiety and depression. In order to further investigate the relationship between anxiety and depression, the current study applied network analysis.

To interpret the mechanisms of any underlying psychopathology and develop effective interventions, it is essential to characterize the interactions between the two different mental disorders. Network models describe mental disorders using an interacting web of symptoms, which can offer new insight into co-occurrence [9, 16]. According to Network Theory, the symptoms of a mental disorder can lead to development of another disorder; the co-occurrence belongs to a dynamic network of symptoms that cause, sustain, and underlie the symptomology [17, 18]. Bridge symptoms can be regarded as the symptoms that connect two mental health disorders, and the activation of the bridge symptoms increase the risk of symptoms transferring from one disorder to another [9]. Thus, the identification of bridge symptoms between depression and anxiety could provide meaningful clinical implications to prevent co-occurrence. This could be done through applying targeted and prioritized treatment for bridge symptoms to control and prevent activation that can lead to the co-occurring symptoms between depression and anxiety. During the pandemic, there has been a dramatic decreases in individuals’ social activities [2]. Considering the preventative measures of quarantine, social distancing, and lockdown, people’s mobile-related activities have been largely reduced. It is likely that motor-related symptoms could then be considered bridge symptoms between anxiety and depression.

To understand how symptoms change over time, several studies have focused on psychologically related distresses during different COVID-19 stages, with a lack of consensus within the studies’ results. In a recent longitudinal study on mental health during COVID-19, no significant changes in anxiety and depression were found in the general Chinese population between the initial outbreak and the after peak period [6]. On the other hand, Qiu et al. [1] conducted a national survey among Chinese individuals and found that the distress caused by COVID-19 decreased significantly over time among the general public. However, the existing studies did not investigate the mechanism and changes in anxiety and depressive symptoms throughout the COVID-19 outbreak and the after peak using network analysis. A recently developed symptom network perspective has highlighted the importance of not only measuring whether symptoms have changed but measuring the interactions between individual symptoms [19–21]. Using network analysis may then provide a more in-depth understanding on the dynamic changes between symptoms of depression and anxiety at different points throughout the pandemic. The researchers aimed to assess the interactions between anxiety and depressive symptom over the outbreak and peak of COVID-19, and to identify the bridge symptoms (i.e., depressive symptoms with strong associations with anxiety symptoms) using network analysis. Considering the COVID-19-related prevention measures of social distancing and isolation, we hypothesized that motor-related symptoms would be the bridge symptoms between depression and anxiety.

## Materials and methods

### Study sample

The current survey included a total of 5274 Chinese participants who completed a surveyed via “Wenjuanxing,” a Chinese online platform providing functions equivalent to Qualtrics. The location was verified by participants’ cellphone GPS trackers. To avoid duplication of data, each IP address was only granted access once to complete the questionnaire.

**Fig. 1:**
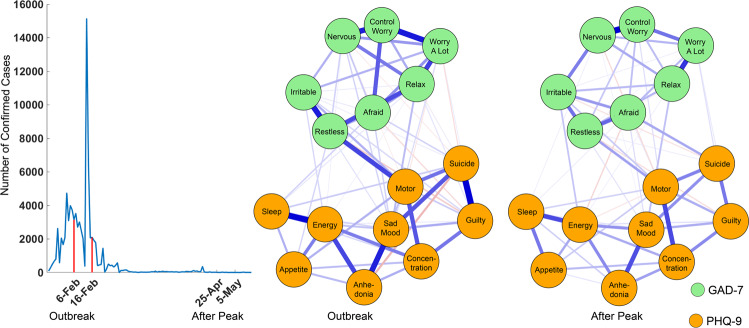
Symptom network at outbreak and after peak stages. The line chat illustrates the data collection periods and the daily confirmed new cases in mainland China. The green nodes denote the GAD-7 items and the orange nodes denote the PHQ-9 items. Meanwhile, the blue edges denote the positive correlations and the red edges denote the negative correlations.

Detailed data collection information, inclusion and exclusion criteria, and demographic information are described in Supplementary information. A total of 5083 participants were included in the analysis. Specifically, 2540 participants (mean age = 25.28 ± 8.07, education years = 15.93 ± 1.82) were surveyed during the outbreak stage from February 6 to 16, 2020 (Fig. 1). And, 2543 participants (mean age = 22.03 ± 6.30, education years = 15.97 ± 1.26) were surveyed during the after peak stage. The study was approved by the ethics committee of Central University of Finance and Economics and The Second Xiangya Hospital of Central South University.

### Assessment of psychopathology symptoms

#### The Patient Health Questionnaire-9 (PHQ-9)

Depression symptoms were assessed via the nine-item PHQ-9 [22]. The items of PHQ-9 and their reference names are listed in Table S1. The scales for the questionnaire are in a four-point Likert format where participants evaluate their symptoms on a scale from 0 (not at all) to 3 (nearly every day), with higher scores indicating severe symptoms. The validated Chinese version uses a cutoff score of 5 to determine whether a participant had mild depression symptoms, and the same cutoff score was used for this study [22–24]. The Cronbach’s alpha was 0.915.

#### The Generalized Anxiety Disorder Scale (GAD-7)

Anxiety symptoms were assessed using the seven-item GAD-7 Scale [25]. The items of GAD-7 and their reference names are listed in Table S1. The scales consist of a four-point Likert format, in which participants evaluate their symptoms on a scale from 0 (not at all) to 3 (nearly every day), with higher scores indicating severe symptoms. The validated Chinese version uses a cutoff score of 5 to determine whether a participant has at least mild anxiety symptoms, and was also used to determine the cutoff score for this study [23, 26, 27]. The Cronbach’s alpha was 0.941.

### Analytical strategies

The changes of sum scores for depression and anxiety were compared, respectively, between the outbreak and after peak stages using two-tailed independent *t*-tests, with the significance level set as 0.05. The network analysis was then performed in the aspects of network estimation, network stability, and network differences [28].

#### Network estimation

In accordance with network parlance, the scores of the items were considered as nodes and the pair-wise correlations between these scores were considered as edges [18, 29–32]. To estimate the symptom network illustrating the relationship between depression and anxiety symptoms, pair-wise Pearson correlations were run and a sparse Gaussian graphical model with the graphical lasso was performed to estimate the network [33]. The tuning parameter was decided upon using the extended Bayesian information criterium [34]. Within this procedure, symptom networks at outbreak and after peak stages were estimated. The R package “bootnet” was utilized to complete this analysis [35]. The network structure was characterized by network centrality indices, this is where each node is placed within a weighted network, i.e., strength, closeness, and betweenness [36, 37]. Specifically, strength is the sum of edge weights directly connected to a node, which measures the importance of a symptom in the network. Closeness is the inverse of the average shortest path length between a node and other nodes, it measures how close the symptom is linked to other symptoms. Betweenness is the number of times that the shortest path between any two nodes passes through another node and measures the importance of the symptom in linking to other symptoms. The “centrality Plot” function from “qgraph” package in R was used to complete this analysis [38]. The role of a symptom as a bridge between anxiety and depressive symptoms was also assessed. Similar to the network centrality, the bridge centrality, which includes bridge strength, bridge closeness, and bridge betweenness, of each symptom was analyzed. The only difference between network and bridge centrality is that the associated two symptoms, as mentioned above, are from different disorders. The bridge centrality of the nodes measures the importance of a symptom in linking two mental health disorders. The complete this analysis the R package “networktools” [39] was used.

#### Network differences

After checking the stability of the network structure (see Supplementary information), the symptom connections and the network properties, as mentioned above, were compared. The comparison was between the outbreak and the after peak stages to allow for any symptom network changes caused by the pandemic to be quantified. The differences were quantified using permutation tests with 1000 iterations [40, 41] using the R package “Network Comparison Test” [42]. Specifically, participants were randomly assigned into two group (within the outbreak group and the same within the after peak group). Then the symptom networks were constructed, estimated, and compared using a bootstrap method of resampling by repeating 1000 times to get the null distribution of the network differences under the null hypothesis. The significance level was set as 0.05.

In addition, the network differences in both edge and network properties, in global and local level, were compared. The global differences in edge weights were measured by the largest difference in paired edges between two networks. Meanwhile, the local edge weight differences were also separately measured. In addition, the global difference in strength was measured by the difference between average strength. Finally, the differences in local network properties were also measured separately.

## Results

### General differences in symptom scores

The severity of each disorder, between outbreak and after peak stages, was compared. It was found that participants at the after peak stage were more depressed than that at the outbreak stage (PHQ-9, *M*_After Peak_ = 4.72, *M*_Outbreak_ = 4.17, *t*_5075.5_ = 4.0313, *p* < 0.001). However, the anxiety disorder scale scores (GAD-7) showed no difference between these two stages (*M*_After Peak_ = 3.60, *M*_Outbreak_ = 3.57). Using the cutoff score of 5 (at least experiencing mild depression and anxiety symptoms), after the peak stage, 42.94% of the participants showed depression symptoms, which is significantly higher (*χ*^2^ = 24.29, *p* < 0.001) than that in outbreak stage (36.14%). Meanwhile, we found more participants showed anxiety symptoms (*χ*^2^ = 10.57, *p* = 0.001) after peak (36.41%), compared to the outbreak stage (32.05%).

### Network estimation and comparison

The estimated networks are displayed in Fig. 1. Detailed edges weights are listed in Tables S2 and S3. The symptom network at outbreak stage showed different patterns regarding the number and thickness of the edges. Before characterizing the network properties and quantifying the property differences, the stability of the symptom networks during outbreak and after peak stages was evaluated by using the bootstrap method, results are displayed in Figs. S1 and 2. These figures showed that most of the edges and centrality were stable. Detailed results are provided in Supplementary information. Therefore, the network differences between the outbreak and after peak stages reflect solid changes of the psychological interaction patterns that were caused by the pandemic.

**Fig. 2:**
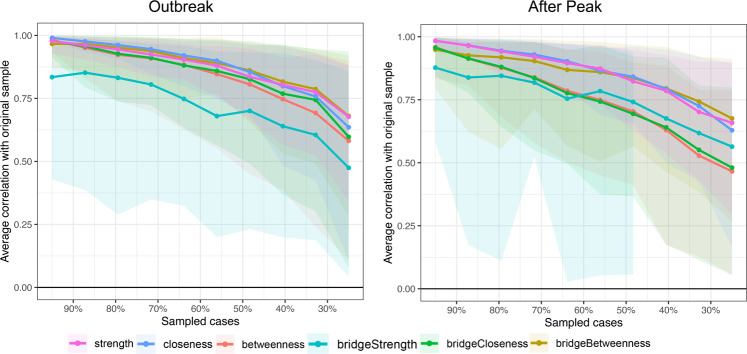
Stability of network structures. The x-axle indicates the included portion of cases, and the y-axle indicates the correlations between the original centrality indices with the estimated centrality after dropping part of the cases. Lines with different colors represent different network properties. The shades indicate the range from the 2.5th quantile to the 97.5th quantile.

The network differences in both edge and network properties were compared. No global differences were found between networks from outbreak and after peak stages. Globally, according to the permutation test, the maximum difference (diff, contrast: after peak − outbreak, same below) between stages in any of the edge weights from both networks was not significant (the maximum difference in Edge was between “afraid” and “inability to relax” symptoms from current networks, diff = −0.16, *p* = 0.20). Meanwhile, the global strength difference between outbreak (global strength = 8.38) and after peak (global strength = 8.24) stages was also found as not significant (*p* = 0.70). However, local differences were found in multiple edges and nodes. Locally, the networks at outbreak and after peak stages differed not only in symptom connections (edge weights), but also in network properties (network and bridge centrality).

**Fig. 3:**
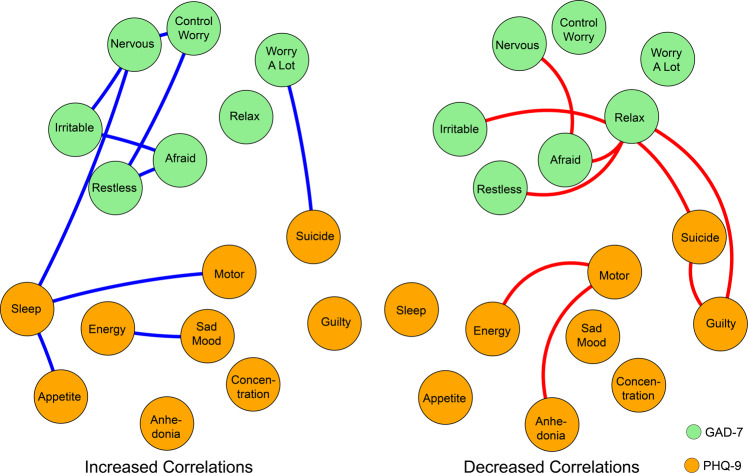
Edges exhibiting significant differences between outbreak and after peak stages. The green nodes denote the GAD-7 items and the orange nodes denote the PHQ-9 items. Meanwhile, the blue edges denote the increased correlations between items at the after peak stage when compared with those in the outbreak stage and the red edges denote the decreased ones.

Specifically, for the edge weights, the significant positive and negative correlations were visualized separately in Fig. 3 (*p* < 0.05). At the after peak stage, insomnia symptom from the PHQ-9 showed stronger connections with impaired motor skills and changes in appetite symptoms from the GAD-7 as well as with nervous symptoms from the PHQ-9. No decreased connections with other symptoms were shown. By contrast, the symptom of inability to relax from the GAD-7 showed a decreased connection with symptoms of being afraid, restless, and irritable from the GAD-7 and also with suicidal thoughts and guilty symptoms from the PHQ-9. There were no increased connections with other symptoms shown. It should also be noted that during the after peak stage, compared to the outbreak stage, suicidal thoughts showed a decreased connection with “inability to relax” and “guilty” symptoms, whereas suicidal thoughts showed an increased connection with the “too much worry” symptom. The decreased connection between feeling guilty and suicidal thoughts from the outbreak stage to the after peak stage is also illustrated in Fig. S1, in which the edge weights, no matter if from the current sample or bootstrapped sample, ranked at the top in the outbreak stage and dropped to number nine in the after peak stage.

**Fig. 4:**
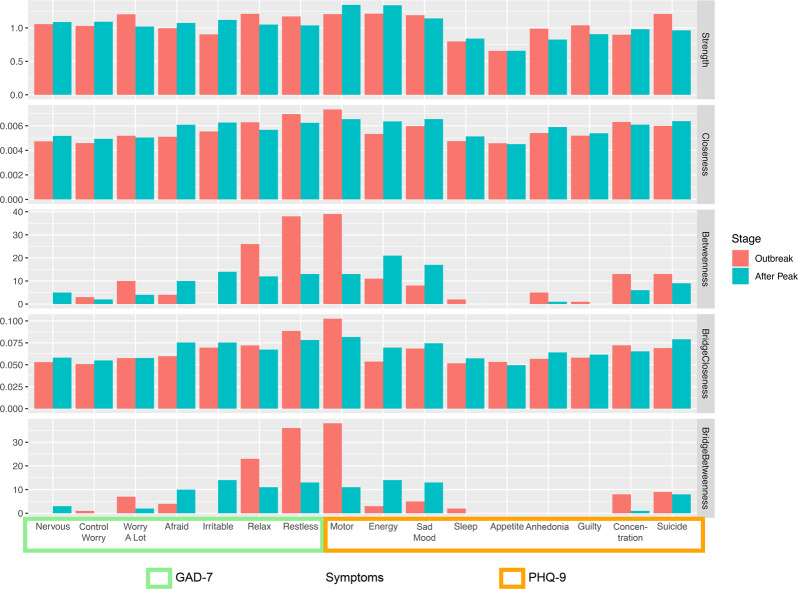
Network and bridge centrality. GAD-7: Generalized Anxiety Disorder-7; PHQ-9: Patient Health Questionnaire-9.

For the network properties, bar plots indicate the network and bridge centrality of each symptom in each stage as displayed in Fig. 4. During the outbreak, psychomotor symptoms such as impaired “motor skills, restless, and inability to relax” exhibited high network betweenness and bridge betweenness. While during the after peak stage, although these symptoms decreased, they were still relatively high when compared with other symptoms. These symptoms might not necessarily exhibit intensive connections with other symptoms. However, they stand between the associated symptoms, which may have played a key role as a mediator that regulated the connections between the symptoms in the network [43]. Moreover, besides these symptoms, several other symptoms also showed increased network and bridge centrality during the after peak stage.

In specific, using permutation tests, it was found that the “inability to relax” symptom showed a decreased strength at the after peak stage (diff = −0.16, *p* = 0.03) when compared to the outbreak stage. Meanwhile, the “restlessness” symptom exhibited decreased betweenness (diff = −25, *p* = 0.04) and the “impaired motor sills symptom” showed decreased betweenness (diff = −26, *p* = 0.01), bridge closeness (diff = −0.021, *p* = 0.048), and bridge betweenness (diff = −27, *p* = 0.01). By contrast, the “irritable” symptom showed increased strength (diff = 0.22, *p* = 0.02), betweenness (diff = 14, *p* = 0.03), and bridge betweenness (diff = 14, *p* = 0.02) during the after peak stage, compared to the outbreak stage. Meanwhile, the “loss of energy” symptom showed increased bridge closeness (diff = 0.016, *p* = 0.03) and bridge betweenness (diff = 11, *p* = 0.02).

## Discussion

The novelty of the current study was to evaluate the psychopathological symptom changes between the outbreak and after peak in China, which have significant implications for other countries that still have not reached their after peak. The current study identified the bridge symptoms and aimed to identify the risks of co-occurrence between anxiety and depressive symptoms during different phrases of COVID-19 to prevent increasing psychological distress. The network differences and changes between outbreak and after peak stages showed the impact of the COVID-19 pandemic on psychological interaction patterns.

The prevalence of anxiety and depression in this study during outbreak was 32.05% and 36.14%, and during after peak phase was 36.41% and 42.94%. Similar to the meta-analysis on depression and anxiety during the COVID-19, over one-third of the population suffered from anxiety and depressive symptoms [8]. Researchers have suggested that the mental health consequences of COVID-19 could last over time and that mental health problems could peak later than the actual pandemic [3]. Our results were consistent with the prediction and showed that after the COVID-19 peak the prevalence of depression and anxiety increased. This could due to the far-reaching influences of COVID-19, such as the induced economic uncertainty, the fear of economic crisis and recession, and increased unemployment [44, 45]. These aftereffects could all work toward increasing anxiety and depression after the actual pandemic peak.

Research has noted that different mental health problems have emerged during the COVID-19 outbreak, which mainly included anxiety and depression [46]. Previous research has examined the symptoms of anxiety and depression using network analysis in psychiatric patients and found that sad mood and worry were the most central symptoms in the network [28]. In the current study, during the outbreak stage psychomotor symptoms such as impaired motor skills, restlessness, and inability to relax were the most central symptoms in the network. During the after peak stage these symptoms showed a decreased centrality but were still relatively high when compared with other symptoms. In addition, the irritable symptom showed increased centrality during the after peak stage. That is, after the peak time, psychomotor centrality decreased, while the mental health problems were more severe due to the contributions from other non-psychomotor-related aspects. After the pandemic peak time, normal social activities started to resume. This could explain why people’s physical- and motor-related activities began to show normality as the psychomotor-related symptoms would be eased.

However, the mental health problems caused by the pandemic could have prolonged effects [3] and people might be anxious and depressed from other non-psychomotor aspects. During the COVID-19 period, there was a perceived decrease in physical-related activities [2], which correspond with the central symptoms identified from the data. Compared with the non-symptomatic group, depressed patients presented disturbances in psychomotor symptoms in terms of motor activities, body movement, and motor reaction time [47–49]. Researchers have proposed that psychomotor symptoms may have unique significance in depression, which could explain the psychomotor manifestations and pathophysiologic significance of depression [47]. Restless-agitation in anxiety is also related to psychomotor functions, in which the higher level of restless-agitation indicated more severe anxiety [25].

After assessing the interactions between anxiety and depressive symptoms, it was identified that the bridge symptoms during the outbreak also focused on psychomotor symptoms such as impaired motor skills, restlessness, and inability to relax. In particular, the impaired motor skill symptoms showed a significant decrease in bridge centrality during the after peak phase, although it was still relatively high when compared with other symptoms. Meanwhile, it was also observed that the inability to relax showed decreased connections with being afraid, restlessness, suicidal thoughts, and feelings of guilt. In addition, during the after peak phase, other bridge symptoms such as irritable and loss of energy emerged, which showed higher bridge centrality than the outbreak stage. In a risky network, the connections among symptoms are tight and strong, and the activation of one symptoms could lead to others, resulting in more severe consequences [28, 30]. During the outbreak and after peak, the occurrence of either impaired motor skills with depression symptoms or restlessness with anxiety symptoms could increase the risk of activation for other mental disorders. This was different from a previous study conducted during the pre-pandemic period. Previous network analysis has shown that the association of anxiety and depression can be attributed to the strong connection from anxious worrying to sleep problems and difficulty concentrating [50]. Our results also indicated that during the after peak insomnia showed enhanced connections with appetite changes, impaired motor skills, and nervous symptoms.

Compared to the non-pandemic period, there have been a wide-scale lockdown and restrictions on transportation during the COVID-19 pandemic. The beneficial effects of physical health on mental health have been well-documented in research [51, 52]. COVID-19 is having a negative impact on people’s physical activity on a global level [53, 54]. Recent COVID-19 research in psychiatric patients also reported that poor physical health was related with higher levels of anxiety and depression [55]. This could explain why the impaired motor skills aspect and restlessness become the bridge symptoms between anxiety and depression.

Depression and anxiety are frequently co-occurring mental disorders, and previous research has indicated the likelihood of a causal relationship between these two mood disorders [50]. A cognitive neuroscience study using default model network (DMN) indicated that cortical areas of the DMN showed functional connectivity associated with anxiety and depression [56]. Similar to previous studies, the current study cannot confirm the causal relation between anxiety and depression. However, the current network analysis can be utilized in clinical practice during the COVID-19 period. A previous study suggested that interventions should focus on depression and anxiety symptoms which are most closely related to other symptoms, since those symptoms should theoretically decrease the associated risk [57]. Moreover, symptoms with a high centrality may also have crucial roles in the network [58]. Those core symptoms could have important roles in maintaining the psychopathology network and treating those symptoms could help to cure the psychopathology. That is, for treating COVID-19-related mood problems, the study results suggest clinical practitioners to focus on the symptoms highlighted by our network analysis.

Researchers have expressed concern about the consequence of mental disorders resulting from the COVID-19 pandemic [59] and mental health professionals have speculated a globe increase of mental disorders due to the impact of COVID-19 [60, 61]. WHO has also mentioned that COVID-19 related specific measures, such as self-isolation, quarantine, and social distancing, might increase loneliness and mood-related problems such as anxiety and depression in people [62]. Our results showed that during the after peak phase, the impaired motor-skill-related symptoms were still prominent. It is hard to predict the duration of the COVID-19 crisis, especially as cities such as Leicester, United Kingdom [63] are undergoing a second lockdown. It is possible that impaired motor-skill-related symptoms could persistent in people in the second lockdown control zones. During the COVID-19 lockdown, physical health professionals have recommended people to stay active with home-based physical activities in order to maintain their health, engaging in activities such as aerobic exercise training and body weight training [53]. A healthy lifestyle and regular exercise are associated with an enhanced immune system [2], which could help protect people from COVID-19-related health problems. This study suggests that health professionals could provide tailored and practical suggestions for the general population by targeting mood symptoms through exercise as a prevention or as a treatment strategy. Researchers have proposed to use mindfulness-based stress reduction practices to improve mental health during the COVID-19 [64–66]. In the current literature, mindfulness-based interventions have shown effectiveness in reducing anxiety and depression [67, 68].

There are several limitations to the study that should be acknowledged. First, depression and anxiety were measured by self-reported questionnaires rather than systematic diagnosis. Second, this network analysis on depression and anxiety focused specifically on the COVID-19 pandemic and cannot be generalized to non-pandemic times. Therefore, the central symptoms and bridge symptoms identified in the current study may not applicable during other periods. Third, the age of the participants was relatively young. Fourth, due to the cross-sectional design, causal relationship could not be established. Future longitudinal studies are needed to investigate the causal relationship between anxiety and depression. Finally, the study did not measure the changes in physical health and the degree of reduction in physical activities during COVID-19.

In conclusion, this is the first network analysis focusing on psychopathological symptoms during the COVID-19 pandemic, which provides valuable insights to understand the interactions between depression and anxiety. The current findings indicated the central symptoms and bridge symptoms during the COVID-19 outbreak and after peak stages in order to provide clinical suggestions for psychological interventions that target reducing the co-occurrence of symptoms between different mental health problems.

## Supplementary information

Appendix
