# Supplementary material for: Changes in network centrality of psychopathology symptoms between the COVID-19 outbreak and after peak
Source: Mol Psychiatry. 2020 Sep 14;25(12):3140–9. doi: 10.1038/s41380-020-00881-6 (PMC7488637; doi:10.1038/s41380-020-00881-6)
Supplement: Supplementary file 1 — Appendix [file 41380_2020_881_MOESM1_ESM.docx]

**Appendix**

***Study Sample***

The first round of data collection was conducted during the outbreak stage from February 6 to 16, 2020, when China reported an increasing number of COVID-19 positive cases with around 2,000 new cases daily ^1^. The second round of data was collected during the after peak stage from April 25 to May 5, 2020, during which the COVID-19 confirmed cases dropped to around 20 new cases or fewer daily. The inclusion criteria were: 1) lived in mainland China during the survey; 2) Aged at least 16 years old. The exclusion criteria were: 1) frontline healthcare workers (e.g., medical staffs, doctors, and nurses); 2) history of mental disorders; 3) COVID-19 infected patients and their family members. This survey took about 10-15 minutes to complete. All participants were informed of the purpose and procedures of this survey, and all of them provided written consent.

A total of 5,083 participants were included in the analysis. The anxiety and depressive symptoms were assessed at outbreak stage and the ease stage. Specifically, 2,540 participants were surveyed during the outbreak stage. There were 71.34% (n =1,812) female, and majority of them were Han ethnicity (88.90%, n = 2, 258). Based on the region division by the National Bureau of Statistics ^2^, there were 31.49% participants who lived in the east (N = 799), 42.53% in the middle (N = 1079), 4.77% in the northeast (N = 121), and 21.21% in the west (N =538) of mainland China. Meanwhile, 2,543 participants were surveyed during the after peak stage.

***Network Stability***

Before testing the network differences, the stability of the network was evaluated in terms of edge weights and network properties. The stability of edge weights was evaluated using a bootstrap with 1000 replicates ^3, 4^. The mean values and 95% confidence intervals of the edge weights of the networks from the bootstrapped sample were plotted together with the edge weights of the networks from the current sample. By sorting the bootstrapped mean edge weights, it can be clearly illustrated whether the edges weights of the current sample are consistent with the bootstrapped ones, and can be used as an indicator of network stability.

Meanwhile, in terms of network properties, the stability of the network structure was illustrated by using a case-dropping subset bootstrap with 1000 replicates. The main procedure of this method is to assess the correlation between the original centrality indices and the estimated centrality, after dropping a certain portion of the cases. If the network structure is stable, then a high correlation coefficient should be observed. Furthermore, the overall stability of the network structure was quantified by using a correlation stability coefficient (CS), which measured the maximum proportion of cases that can be dropped, such that with 95% probability the correlation would reach a certain value (0.7 in current study, cor = 0.7). It is suggested that CS_cor = 0.7_ should not be below 0.25 and better if it is above 0.5 ^5^. The bootstrap procedure and the overall stability were calculated using the *bootnet* R package.

***Results of Network Stability***

The edge weights in the current sample were largely consistent with the bootstrapped sample, especially for the connections with larger weights, which primarily indicated a stable network structure of the current symptom networks.

The bridge strength of the network was extremely unstable at both stages (for both stages, CS_cor=0.7_ = 0). In addition, symptom networks at the after peak stage exhibited less stable structures, as its betweenness (CS_cor=0.7_ = 0.21) and bridge closeness (CS_cor=0.7_ = 0.21) showed a CS lower than 0.25. Therefore, the bridge strength was not included in any further analysis and any interpretations regarding the betweenness and bridge closeness of the symptom network at after peak stage should be taken with great care. Besides that, the symptom network at outbreak stage exhibited a relatively stable structure with the CS of strength (CS_cor=0.7_ = 0.52) and closeness (CS_cor=0.7_ = 0.52) exceeding 0.5, and the CS of other metrics also exceeding0.25 (for CS_cor=0.7_, betweenness = 0.36, bridge closeness = 0.28, bridge betweenness = 0.44). Using 30% of the cases, the average correlations within the original sample exceeded 0.5 for both stages, which indicated that the symptom network preserved a stable structure regardless of the epidemic stages.

**Figure S1.** Stability of Edge Weights


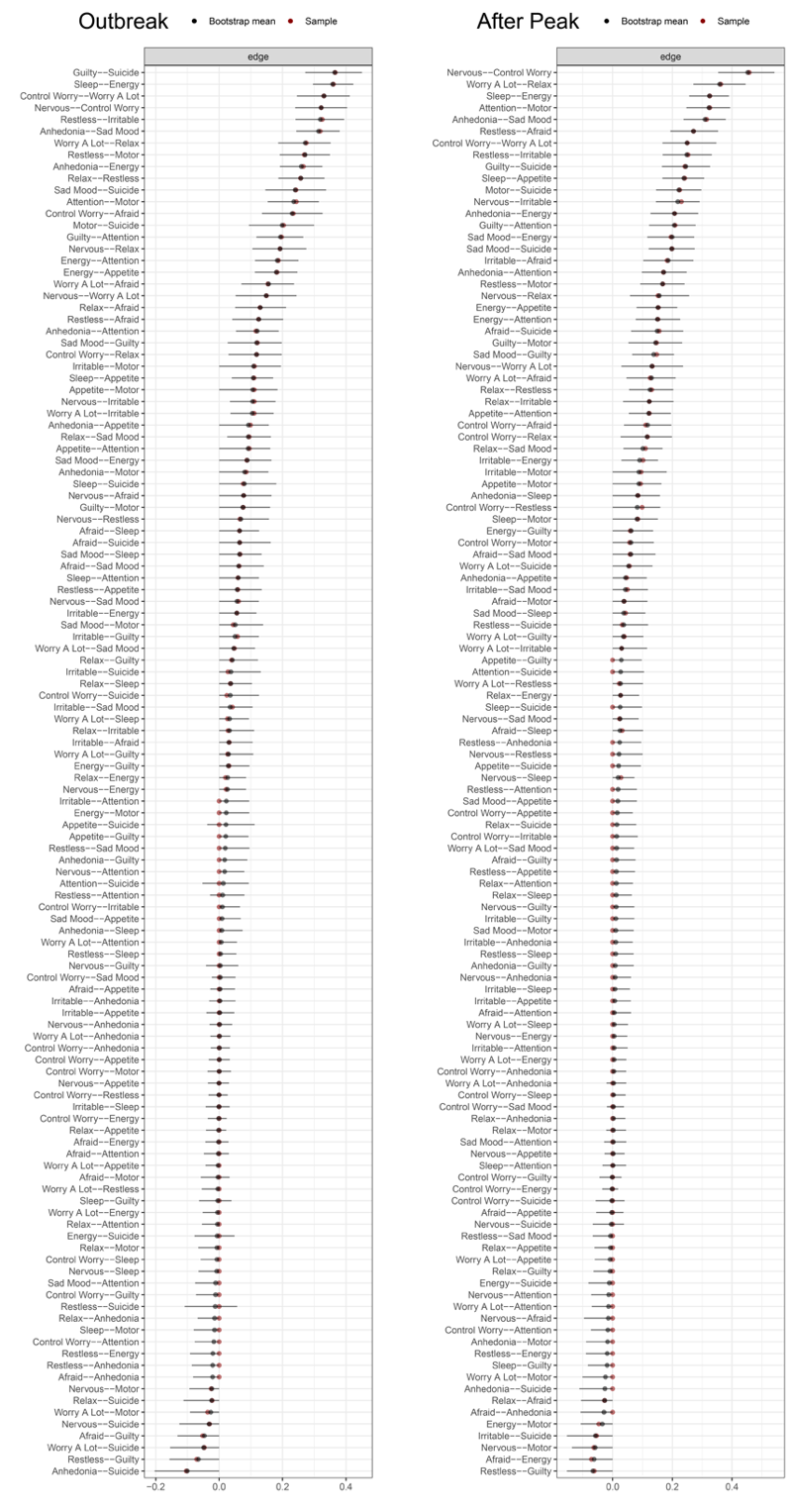


*Note: The x-axle indicates the edge weights and the y-axle indicates the nodes linked by the edges. The black dots denote the mean value of the bootstrapped edge weights and the red dots denote the edge weights from current sample. The black lines denote the 95% confidence intervals of the bootstrapped sample.*

Table S1. Questionnaires and Items Used for the Assessment of psychopathology symptoms

|  | Reference Name | Item |
| --- | --- | --- |
| PHQ-9 | Anhedonia | Little interest or pleasure in doing things |
|  | Sad Mood | Feeling down, depressed, or hopeless |
|  | Sleep | Trouble falling or staying asleep, or sleeping too much |
|  | Energy | Feeling tired or having little energy |
|  | Appetite | Poor appetite or overeating |
|  | Guilty | Feeling bad about yourself - or that you are a failure or have let yourself or your family down |
|  | Concentration | Trouble concentrating on things, such as reading the newspaper or watching television |
|  | Motor | Moving or speaking so slowly that other people could have noticed Or the opposite - being so fidgety or restless that you have been moving around a lot more than usual |
|  | Suicide | Thoughts that you would be better off dead, or of hurting yourself in some way |
| GAD-7 | Nervous | Feeling nervous, anxious or on edge |
|  | Control Worry | Not being able to stop or control worrying |
|  | Worry A Lot | Worrying too much about different things |
|  | Relax | Trouble relaxing |
|  | Restless | Being so restless that it is hard to sit still |
|  | Irritable | Becoming easily annoyed or irritable |
|  | Afraid | Feeling afraid as if something awful might happen |

*PHQ-9: Patient Health Questionnaire-9; GAD-7: Generalized Anxiety Disorder Scale*

*Table S2. Estimated Edge Weights during the Outbreak*

|  | Nervous | Control Worry | Worry A Lot | Relax | Restless | Irritable | Afraid | Anhedonia | Sad Mood | Sleep | Energy | Appetite | Guilty | Concentration | Motor | Suicide |
| --- | --- | --- | --- | --- | --- | --- | --- | --- | --- | --- | --- | --- | --- | --- | --- | --- |
| Nervous | NA | 0.32 | 0.15 | 0.19 | 0.07 | 0.11 | 0.08 | NA | 0.06 | NA | 0.02 | NA | NA | NA | -0.03 | -0.03 |
| Control Worry | 0.32 | NA | 0.33 | 0.12 | NA | NA | 0.23 | NA | NA | NA | NA | NA | NA | NA | NA | 0.02 |
| Worry A Lot | 0.15 | 0.33 | NA | 0.28 | NA | 0.11 | 0.15 | NA | 0.05 | 0.03 | NA | NA | 0.03 | NA | -0.04 | -0.05 |
| Relax | 0.19 | 0.12 | 0.28 | NA | 0.26 | 0.03 | 0.13 | NA | 0.09 | 0.04 | 0.02 | NA | 0.04 | NA | NA | -0.02 |
| Restless | 0.07 | NA | NA | 0.26 | NA | 0.33 | 0.12 | NA | NA | NA | NA | 0.06 | -0.07 | NA | 0.27 | NA |
| Irritable | 0.11 | NA | 0.11 | 0.03 | 0.33 | NA | 0.03 | NA | 0.04 | NA | 0.06 | NA | 0.06 | NA | 0.11 | 0.03 |
| Afraid | 0.08 | 0.23 | 0.15 | 0.13 | 0.12 | 0.03 | NA | NA | 0.06 | 0.06 | NA | NA | -0.05 | NA | NA | 0.07 |
| Anhedonia | NA | NA | NA | NA | NA | NA | NA | NA | 0.32 | NA | 0.27 | 0.10 | NA | 0.12 | 0.09 | -0.10 |
| Sad Mood | 0.06 | NA | 0.05 | 0.09 | NA | 0.04 | 0.06 | 0.32 | NA | 0.07 | 0.09 | NA | 0.12 | NA | 0.04 | 0.24 |
| Sleep | NA | NA | 0.03 | 0.04 | NA | NA | 0.06 | NA | 0.07 | NA | 0.36 | 0.11 | NA | 0.06 | NA | 0.08 |
| Energy | 0.02 | NA | NA | 0.02 | NA | 0.06 | NA | 0.27 | 0.09 | 0.36 | NA | 0.18 | 0.03 | 0.19 | NA | NA |
| Appetite | NA | NA | NA | NA | 0.06 | NA | NA | 0.10 | NA | 0.11 | 0.18 | NA | NA | 0.10 | 0.11 | NA |
| Guilty | NA | NA | 0.03 | 0.04 | -0.07 | 0.06 | -0.05 | NA | 0.12 | NA | 0.03 | NA | NA | 0.20 | 0.08 | 0.37 |
| Concentration | NA | NA | NA | NA | NA | NA | NA | 0.12 | NA | 0.06 | 0.19 | 0.10 | 0.20 | NA | 0.24 | NA |
| Motor | -0.03 | NA | -0.04 | NA | 0.27 | 0.11 | NA | 0.09 | 0.04 | NA | NA | 0.11 | 0.08 | 0.24 | NA | 0.20 |
| Suicide | -0.03 | 0.02 | -0.05 | -0.02 | NA | 0.03 | 0.07 | -0.10 | 0.24 | 0.08 | NA | NA | 0.37 | NA | 0.20 | NA |

*Table S3. Estimated Edge Weights After the Peak*

|  | Nervous | Control Worry | Worry A Lot | Relax | Restless | Irritable | Afraid | Anhedonia | Sad Mood | Sleep | Energy | Appetite | Guilty | Concentration | Motor | Suicide |
| --- | --- | --- | --- | --- | --- | --- | --- | --- | --- | --- | --- | --- | --- | --- | --- | --- |
| Nervous | NA | 0.46 | 0.13 | 0.15 | NA | 0.23 | NA | NA | 0.02 | 0.03 | NA | NA | NA | NA | -0.06 | NA |
| Control Worry | 0.46 | NA | 0.25 | 0.12 | 0.10 | NA | 0.11 | NA | NA | NA | NA | NA | NA | NA | 0.06 | NA |
| Worry A Lot | 0.13 | 0.25 | NA | 0.36 | 0.02 | 0.03 | 0.13 | NA | NA | NA | NA | NA | 0.04 | NA | NA | 0.06 |
| Relax | 0.15 | 0.12 | 0.36 | NA | 0.13 | 0.12 | -0.03 | NA | 0.11 | NA | 0.03 | NA | NA | NA | NA | NA |
| Restless | NA | 0.10 | 0.02 | 0.13 | NA | 0.25 | 0.27 | NA | NA | NA | NA | NA | -0.06 | NA | 0.17 | 0.03 |
| Irritable | 0.23 | NA | 0.03 | 0.12 | 0.25 | NA | 0.18 | NA | 0.05 | NA | 0.10 | NA | NA | NA | 0.10 | -0.05 |
| Afraid | NA | 0.11 | 0.13 | -0.03 | 0.27 | 0.18 | NA | NA | 0.06 | 0.03 | -0.07 | NA | NA | NA | 0.04 | 0.16 |
| Anhedonia | NA | NA | NA | NA | NA | NA | NA | NA | 0.32 | 0.09 | 0.21 | 0.05 | NA | 0.17 | NA | NA |
| Sad Mood | 0.02 | NA | NA | 0.11 | NA | 0.05 | 0.06 | 0.32 | NA | 0.04 | 0.20 | NA | 0.15 | NA | NA | 0.20 |
| Sleep | 0.03 | NA | NA | NA | NA | NA | 0.03 | 0.09 | 0.04 | NA | 0.33 | 0.24 | NA | NA | 0.08 | NA |
| Energy | NA | NA | NA | 0.03 | NA | 0.10 | -0.07 | 0.21 | 0.20 | 0.33 | NA | 0.15 | 0.06 | 0.15 | -0.05 | NA |
| Appetite | NA | NA | NA | NA | NA | NA | NA | 0.05 | NA | 0.24 | 0.15 | NA | NA | 0.12 | 0.09 | NA |
| Guilty | NA | NA | 0.04 | NA | -0.06 | NA | NA | NA | 0.15 | NA | 0.06 | NA | NA | 0.21 | 0.15 | 0.24 |
| Concentration | NA | NA | NA | NA | NA | NA | NA | 0.17 | NA | NA | 0.15 | 0.12 | 0.21 | NA | 0.33 | NA |
| Motor | -0.06 | 0.06 | NA | NA | 0.17 | 0.10 | 0.04 | NA | NA | 0.08 | -0.05 | 0.09 | 0.15 | 0.33 | NA | 0.22 |
| Suicide | NA | NA | 0.06 | NA | 0.03 | -0.05 | 0.16 | NA | 0.20 | NA | NA | NA | 0.24 | NA | 0.22 | NA |

References

1. Wu Z, McGoogan JM. Characteristics of and important lessons from the coronavirus disease 2019 (COVID-19) outbreak in China: summary of a report of 72 314 cases from the Chinese Center for Disease Control and Prevention. *Jama* 2020; **323**(13)**:** 1239-1242.

2. Protocal for region division. <http://www.stats.gov.cn/ztjc/zthd/sjtjr/dejtjkfr/tjkp/201106/t20110613_71947.htm>, 2011, Accessed Date. 2 Sep 2020

3. van Borkulo C, Boschloo L, Borsboom D, Penninx BW, Waldorp LJ, Schoevers RA. Association of Symptom Network Structure With the Course of Depression. *JAMA Psychiatry* 2015; **72**(12)**:** 1219-1226.

4. Curtiss J, Ito M, Takebayashi Y, Hofmann SG. Longitudinal network stability of the functional impairment of anxiety and depression. *Clinical Psychological Science* 2018; **6**(3)**:** 325-334.

5. Epskamp S, Borsboom D, Fried EI. Estimating psychological networks and their accuracy: A tutorial paper. *Behavior Research Methods* 2018; **50**(1)**:** 195-212.
